# Impairment of Mitochondrial Redox Status in Peripheral Lymphocytes of Multiple Sclerosis Patients

**DOI:** 10.3389/fnins.2019.00938

**Published:** 2019-09-04

**Authors:** Hugo Gonzalo, Lara Nogueras, Anna Gil-Sánchez, José Vicente Hervás, Petya Valcheva, Cristina González-Mingot, Meritxell Martin-Gari, Marc Canudes, Silvia Peralta, Maria José Solana, Reinald Pamplona, Manuel Portero-Otin, Jordi Boada, Jose Carlos Enrique Serrano, Luis Brieva

**Affiliations:** ^1^Institut de Recerca Biomèdica de Lleida, Lleida, Spain; ^2^Clinical University Hospital of Valladolid (HCUV), Department of Research and Innovation, SACYL/IECSCYL, Valladolid, Spain; ^3^Universitat de Lleida, Departament de Medicina Experimental, Lleida, Spain; ^4^Hospital Universitario Arnau de Vilanova, Lleida, Spain

**Keywords:** multiple sclerosis, oxidative stress, mitochondria, superoxide anion, mitochondrial complexes

## Abstract

Literature suggests that oxidative stress (OS) may be involved in the pathogenesis of multiple sclerosis (MS), in which the immune system is known to play a key role. However, to date, the OS in peripheral lymphocytes and its contribution to the disease remain unknown. The aim of the present study was to explore the influence of OS in peripheral lymphocytes of MS patients. To that end, a cross-sectional, observational pilot study was conducted [*n* = 58: 34 MS and 24 healthy subjects (control group)]. We have measured superoxide production and protein mitochondrial complex levels in peripheral blood mononuclear cells (PBMCs) isolated from MS patients and control. Lactate levels and the antioxidant capacity were determined in plasma. We adjusted the comparisons between study groups by age, sex and cell count according to case. Results demonstrated that PBMCs, specifically T cells, from MS patients exhibited significantly increased superoxide anion production compared to control group (*p* = 0.027 and *p* = 0.041, respectively). Increased superoxide production in PBMCs was maintained after the adjustment (*p* = 0.044). Regarding mitochondrial proteins, we observe a significant decrease in the representative protein content of the mitochondrial respiratory chain complexes I-V in PBMCs of MS patients (*p* = 0.002, *p* = 0.037, *p* = 0.03, *p* = 0.044, and *p* = 0.051, respectively), which was maintained for complexes I, III, and V after the adjustment (*p* = 0.026; *p* = 0.033; *p* = 0.033, respectively). In MS patients, a trend toward increased plasma lactate concentration was detected [8.04 mg lactate/dL (5.25, 9.49) in the control group, 11.36 mg lactate/dL (5.41, 14.81) in MS patients] that was statistically significant after the adjustment (*p* = 0.013). This might be indicative of compromised mitochondrial function. Finally, antioxidant capacity was also decreased in plasma from MS patients, both before (*p* = 0.027) and after adjusting for sex and age (*p* = 0.006). Our findings demonstrate that PBMCs of MS patients show impaired mitochondrial redox status and deficient antioxidant capacity. These results demonstrate for the first time the existence of mitochondrial alterations in the cells immune cells of MS patients already at the peripheral level.

## Introduction

Multiple sclerosis (MS) is an inflammatory, demyelinating, and neurodegenerative disease of the central nervous system (CNS) (Reich et al., 2018). Also, it is currently considered the most disabling chronic neurological disease in young adults (Nylander and Hafler, 2012). Genetic susceptibility has been postulated as a possible factor (Sadovnick et al., 1993; Dyment et al., 2004) that, combined with environmental factors (Ascherio and Munger, 2010; Hewer et al., 2013; Pender and Burrows, 2014), can activate a cascade of inflammatory events responsible for the autoimmune response. However, its etiology remains to be understood (Chastain and Miller, 2012).

An increasing number of studies have shown that, oxidative stress (OS) — defined as the homeostatic imbalance between the production of free radicals and their neutralization by antioxidant defenses (Salim, 2017) – plays an important role in the pathogenesis of MS (Ridet et al., 1997; Gilgun-Sherki et al., 2004; Sospedra and Martin, 2005; Miljković and Spasojević, 2013; Ohl et al., 2016). So far, OS has been shown to be involved in different MS processes such as: (i) in the activation of immune cells, especially T cells (Corthay, 2006); (ii) in the loss of blood – brain barrier (BBB) selectivity (Wu and Tsirka, 2009), (iii) facilitating T-cell migration and infiltration into the CNS (Marui et al., 1993), (iv) increasing the expression of the cytokine network (Kroenke et al., 2008; Jäger et al., 2009), (vi) in MS tissue spread, especially mediated by lipooxidation processes (Gonzalo et al., 2012; Nogueras et al., 2019); and (vii) promoting changes in the clinical evolution of the disease (Fiorini et al., 2013).

At the cellular level, reactive oxygen species (ROS) directly alter the CNS myelin-producing cells: the oligodendrocytes and especially their progenitors, the oligodendrocyte precursor cells (OPCs) (Back et al., 2002; Fünfschilling et al., 2012). These phenomena considerably affect not only the demyelination but also the remyelination process. On the one hand, ROS play a destructive role in demyelinating disorders causing death of mature oligodendrocytes by apoptosis (Cai and Xiao, 2016). This phenomenon facilitates the loss of existing myelin/oligodendrocytes and subsequent demyelination process. On the other, ROS also disrupts OPCs maturation (French et al., 2009; Cunniffe and Coles, 2019) limiting the remyelination process. Both mechanisms result in the axonal damage, the main cause of neurodegeneration in MS (Lee et al., 2014). These aspects are mainly studied at CNS level, in order to establish prognostic biomarkers of clinical evolution. However, most of the studies are performed in experimental models, and very few are validated in MS patients.

At systemic level, studies exploring the involvement of OS in MS are limited and sometimes difficult to interpret. The plasma of MS patients has been reported to have a lower antioxidant capacity (Karlík et al., 2015) and higher levels of oxidative damage to both lipids (Toshniwal and Zarling, 1992) and proteins (Oliveira et al., 2012). This suggests that these phenomena are typical for the CNS but, as the BBB is compromised, it can spread systemically.

Mitochondria are present in all eukaryotic cells. Their main function is to provide energy in ATP form by oxidation, with ROS generation as an associated phenomenon. The first ROS to be generated is the superoxide anion, produced mainly by electron leakage in complexes I and III of the electron transport chain (Turrens, 2003). The conversion of superoxide anion to hydrogen peroxide is rapid due to the action of superoxide dismutase. Nevertheless, superoxide anion is a very reactive ROS, since its reaction with proteins, DNA and lipids occurs within a few microseconds, causing harmful effects on cells, or reacting with other elements to create and spread new ROS (Halliwell and Gutteridge, 2007).

The role of mitochondria in the pathogenesis of MS was suggested following the observation that patients with typical mutations of Leber’s hereditary optic neuropathy, a disease caused by mutations in mitochondrial DNA (Wallace et al., 1988), had a phenotype indistinguishable from that of MS (Olsen et al., 1995). Since then, several types of mitochondrial dysfunction have been described in MS patients. Specifically, Mahad’s team reported mitochondrial dysfunction in the cortex and white matter (Mahad et al., 2008), as well as defects in the mitochondrial respiratory chain in neuronal axons, oligodendrocytes and astrocytes (Mahad et al., 2009). Besides, MS-specific mutations cause clonal expansion of mitochondrial DNA deletions (Larsson, 2010), as well as a decrease in the efficiency of oxidative phosphorylation (Haider, 2015) together with an increase in ROS production (Yakes and Van Houten, 1997; Campbell et al., 2014). All these aspects have been demonstrated in different cell lines at CNS level, but very few studies have been conducted at systemic level, and none to date has focused on the study of circulating immune cells. Consequently, the objective of this work is to study the peripheral blood mononuclear cells (PBMC) under the hypothesis that there is an altered mitochondrial situation at peripheral level in the immune cells of MS patients.

To further characterization of the lymphocyte redox status, we evaluated the content of representative peptides from mitochondrial respiratory complexes, their physiological effect on ROS production as well as compared these variables with plasma antioxidant capacity.

The results provide not only a new pathological mechanism of the heterogeneous MS, which would help to elucidate the lack of therapeutic response by some patients (patients with suboptimal response to treatment), but also the basis for development of novel MS biomarkers, useful for diagnosis, prognosis, and therapeutic response.

## Materials and Methods

### Study Design

This cross-sectional, observational pilot study included a total of 58 participants: 34 patients diagnosed with the relapsing-remitting form of MS according to the 2010 revised McDonald criteria (Polman et al., 2011) and 24 sex and age matched healthy individuals (control group). This study follows the Code of Ethics of the World Medical Association (Declaration of Helsinki). The local medical ethics committee of the Arnau de Vilanova University Hospital (Lleida, Spain) approved the study. All participants signed an informed consent and IRB Lleida Biobank (B.0000682) and PLATAFORMA BIOBANCOS PT13/0010/0014 preserved the samples in optimal conditions. Clinical variables analyzed were: age, sex, disability status (EDSS, expanded disability status scale), disease duration, and treatments.

### Isolation of Peripheral Blood Mononuclear Cells

Peripheral blood mononuclear cells were isolated from peripheral blood obtained by standard venipuncture using Ficoll^®^ (lymphocyte separation medium, BioWhittaker, Lonza, Barcelona, Spain) according to a previously published method (Murray and Rajeevan, 2013). Briefly, 2 × 10 mL of EDTA anticoagulated blood were centrifuged at 300 *g* for 10 min and the top 3 mL containing plasma was separated and stored at −20°C for other assays. The remaining blood was diluted with an equal volume of phosphate-buffered saline (PBS), pH 7.4. The resulting blood-PBS mixture was added to Ficoll^®^ (2:1, v: v) and centrifuged at 460 *g* for 30 min at room temperature with the lowest acceleration and brake program. The PBMC interface was carefully removed by pipetting and washed with PBS. Afterward, a centrifugation at 300 *g* for 10 min take place. Cell count and viability was determined using a Neubauer chamber. We used trypan blue for non-viable cells identification, and cell viability was calculated using the total cell count and the non-viable cell count. We divided cells into aliquots for flow cytometry or stored at −190°C for further analyses.

### Lymphocyte Population Analyses and Superoxide Production

The lymphocyte population was selected by flow cytometry from the PBMC pool following a previously published method (Ruiz-Argüelles and Pérez-Romano, 2001). Cell debris, as represented by distinct low forward and side scatter were gated out for analysis. First, allophycocyanin (APC)-anti-human CD3 (#3A1, Inmunostep, Salamanca, Spain) (1:5) and CF^TM^ Blue-anti-human CD45 (#20F2, Inmunostep, Salamanca, Spain) (1:20) were added to cells and incubated in the dark for 15 min as recommended by the manufacturer. The sample was washed twice with PBS and centrifuged for 5 min at 4500 rpm. Next, 5 μM MitoSox^TM^ (Red mitochondrial superoxide indicator, Molecular Probes, Invitrogen, Barcelona, Spain) was added to obtain a final concentration of 2.5 μM and incubated for 10 min in the dark. The cells were then washed with PBS and centrifuged again as indicated above. Finally, cells were resuspended in 120 μl PBS containing 0.5% BSA and analyzed through the flow cytometer (digital analyzer cytometer FACS-Canto II). T cells were identified as the population positive for CD45 and CD3 markers (CD45^+^ CD3^+^), with CD45^+^ CD3^–^ cells considered mainly B lymphocytes, to a lesser extent, monocytes. We also identified other PBMCs as double negative markers (CD45^–^ CD3^–^). The MitoSOX probe is widely used and validated for detecting selective superoxide in the mitochondria of viable cells (Robinson et al., 2006), and its binding to superoxide can be quantified by flow cytometry in relation to the median fluorescence intensity (MFI).

### ABTS and Plasma Uric Acid and Lactate Measurement

The antioxidant capacity of plasma was measured as previously indicated (Re et al., 1999). The 2,2′-azino-bis (3-ethylbenzothiazoline-6-sulphonic acid) (ABTS, Sigma-Aldrich, St. Louis, MO, United States) was dissolved with 2.45 mM potassium persulfate to make a final concentration of 7 mM. The ABTS radical cation was produced by reacting ABTS stock solution with 2.45 mM potassium persulfate and leaving it to stand in the dark at room temperature overnight. Trolox (6-hydroxy-2,5,7,8-tetramethychroman-2-carboxylic acid; Aldrich Chemical Co., Gillingham, Dorset, United Kingdom) was used to construct a standard curve. After adding ABTS solution (1:100), absorbance was measured at 405 nm, at 0–6 min. Results were expressed as the Trolox equivalent antioxidant capacity (TEAC) of each sample.

Plasma uric acid and lactate concentrations were measured using a Spinreact Kit (Spinreact, La Valld’en Bas, Girona, Spain) following the manufacturer’s recommendations.

### Analysis of Mitochondrial Respiratory Complexes

After thawing, PBMCs were homogenized in RIPA buffer containing the following protease inhibitors: NaVO_4_ 1 mM, NaF 1 mM, DTPAC 1 mM, and BHT 1 mM (80-6501-23, Amersham Biosciences, Little Chalfont, United Kingdom) as described previously. Protein content was determined using the Bradford assay (Bio-Rad Protein Assay, Hercules, CA, United States). Samples containing 20 μg of proteins were mixed with a standard Laemlli reducing buffer and denatured by heating at 95°C for 3 min (Termobloc Selecta, Barcelona, Spain). Proteins (20 μg) were separated on 12% SDS-polyacrylamide gels at 15 mA/gel. For immunodetection, proteins were transferred using a Mini Trans-Blot Transfer Cell (Bio Rad) in a buffer containing 25 mM Tris, 192 mM glycine, and 20% methanol to polyvinylidene difluoride (PVDF) membranes (Immobilon-P Millipore, Bedford, MA, United States), which were then immersed in blocking solution (0.2% I-Block Tropix AI300, 0.1% Tween in PBS) for 1 h at room temperature. Thereafter, membranes were probed overnight at 4°C with primary antibody cocktail anti-human Total OxPhos Complex Kit (dilution 1:500 in Tris–buffered saline, # 458199, Thermo Fisher Scientific), containing primary antibodies against complex I (18 kDa), complex II (29 kDa), complex III (core 2; 48 kDa), complex IV [cytochrome c oxidase (COX) II subunit, 22 kDa] and F1FO ATPase (F1α; 45 kDa) subunits. As a molecular weight pattern, we use a commercial standard consisting in a mitochondrial extract of a human heart tissue that indicates the location of the complexes to study (2 μg/lane, ref. Ab110337, Abcam).

We use an antibody detecting porin (1:1000, ref. Ab15895, Abcam) for protein normalization. The membrane was washed three times in Tris–buffered saline containing 0.05% Tween 20 and incubated for 1 h at room temperature with the appropriate secondary antibodies (ECL Anti-mouse IgG, horseradish peroxidase-linked whole antibody NA93IV GE Healthcare (1:5000 in Tris–buffered saline)]. After five washes with Tris–buffered saline containing 0.05% Tween 20, bands were visualized by using an enhanced chemiluminescent horseradish peroxidase substrate (Millipore). Signal quantification and recording was performed using the ChemiDoc imaging system (Bio-Rad).

### Statistical Analyses

The study variables were described by means and standard deviations (SD), except for those with non-normal distribution (evaluated by the Shapiro-Wilk test), for which medians and interquartile ranges (IR) were obtained. Non-adjusted differences between groups were analyzed with the Student’s *t*-test, or with the Mann-Whitney *U* test if they showed non-normal distribution. The adjusted mean difference (MS group vs. control group) and 95% confidence interval (CI) for each study outcome were obtained using multivariable linear regression, adjusting for sex and age. Differences in superoxide quantification and antioxidant plasma capacity were additionally adjusted for cell count and uric acid concentration, respectively. All tests were two-sided, at a significance level of 5%. All analyses were carried out with the statistical software R (R Core Team, 2018, version 3.5.2).

## Results

### Clinical and Demographic Characteristics of Participants

Table 1 shows the clinical and demographic characteristics of the participants included in the study. Neither the percentage of women, nor the mean age of volunteers showed significant differences between groups.

**TABLE 1:** Clinical and demographic characteristics of MS patients and controls.

**Characteristics**	**CTL (*n* = 24)**	**MS (*n* = 34)**	***p*-value**
Female, *n* (%)	15 (62.5)	22 (64.7)	>0.999
Age (years), mean (SD)	40.0 (11.2)	44.4 (10.8)	0.145
Age range	22–64	24–65	
EDSS, median (IR)	n/a	1 (0, 3)	
EDSS range	n/a	0–7	
Disease duration, median (IR)	n/a	10 (6, 17)	
Line of therapy, *n* (%)			
No therapy		8 (23.5)	
First line therapy	n/a	16 (47.1)	
Second line therapy		10 (29.4)	

*First line therapy includes glatiramer acetate, interferons, and dimethyl fumarate. Second line therapy includes natalizumab, fingolimod. CTL, control group; EDSS, expanded disability status scale; MS, multiple sclerosis group; SD, standard deviation; IR, interquartile range; n/a, not applicable. Missing values [variable (n)]: EDSS (2); disease duration (2).*

### PBMCs From MS Patients Produce More Superoxide Than the Control Group

**FIGURE 1:**
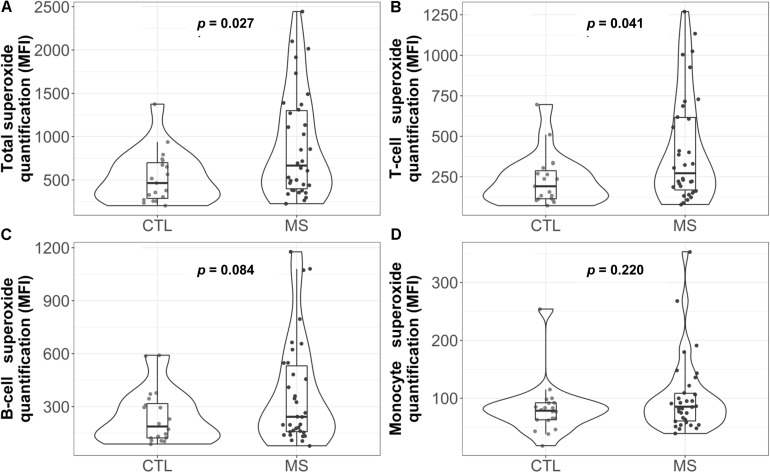
Violin plot and boxplot of superoxide quantification in different white blood cell populations: **(A)** total, **(B)** T cells, **(C)** B cells, and **(D)** monocytes. Dots represent individual data. *P*-values correspond to the Mann-Whitney *U* test. CTL, control group; MS, multiple sclerosis group; MFI=median fluorescence intensity.

As a first approach to assess lymphocyte OS, the possible functional differences in one of the major mitochondrial ROS, the superoxide anion, was analyzed by flow cytometry. Figure 1A shows the total superoxide production by PBMC isolated from the peripheral blood. As can be observed, the MS group showed a statistically significant increase in superoxide production (*p* = 0.027). When mean differences were adjusted for sex, age and cell count, this increase remained statistically significant (Supplementary Table S1).

Analysis of the superoxide production by each cell subpopulation (Figures 1B–D) showed that, in MS patients, T cells presented a higher median concentration of this anion [272 (169, 616.2) in the MS group vs. 192 (114, 287.5) in the control group, *p* = 0.041]. However, these results were not statistically significant when data were adjusted for sex, age and cell count (Supplementary Table S1).

### Mitochondrial Complex Proteins Are Diminished in MS Patients

**FIGURE 2:**
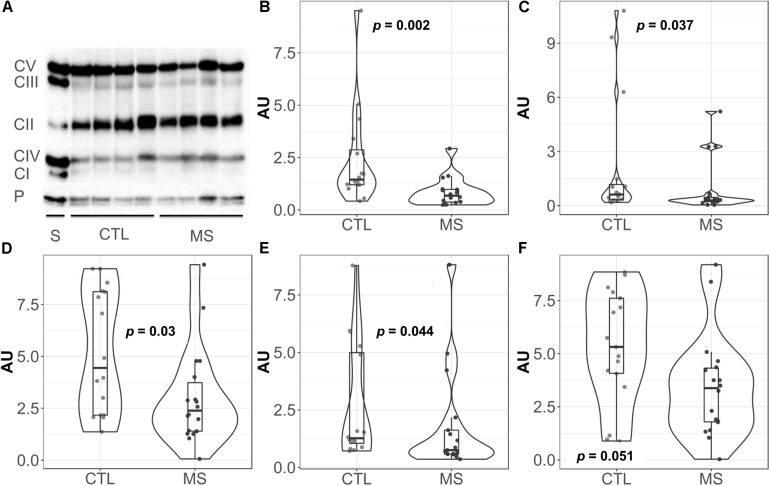
Violin plots and boxplots of data expressed in arbitrary units (AU) corrected for porin. **(A)** Representative western bolt analyses of equal amounts of protein. **(B)** NADH dehydrogenase or complex I. **(C)** Succinate dehydrogenase or complex II. **(D)** coenzyme Q: cytochrome c – oxidoreductase or complex III. **(E)** Cytochrome c oxidase or complex IV. **(F)** ATP synthase or complex V. **(F)** Dots represent individual data. CTL, controls; MS, multiple sclerosis; CI, complex I; CII, complex II; CIII, complex III; CIV, complex IV; CV, complex V; P, porin; S, commercial standard of human heart tissue that indicates the location of the complexes to study.

Given the key role of mitochondria in superoxide production (Boveris and Chance, 1973; Forman and Boveris, 1982), we decided to focus our attention on this organelle. Thus, we analyzed the levels of representative proteins of mitochondrial respiratory chain complexes I-V by western blot (Figure 2A). Quantification revealed statistically significantly lower mean levels of all complexes I–V in MS patients compared to the control group (Figures 2B–F). However, when mean differences were adjusted for age and sex, these differences were only maintained for complex I [−1.48 (−2.72, −0.25), *p* = 0.026], complex III [−1.99 (−3.73, −0.24), *p* = 0.033], and complex V [−1.94 (−3.65, −0.24), *p* = 0.033] (Supplementary Table S1).

### MS Patients Show a Trend to Increase Plasma Lactate Concentration

**FIGURE 3:**
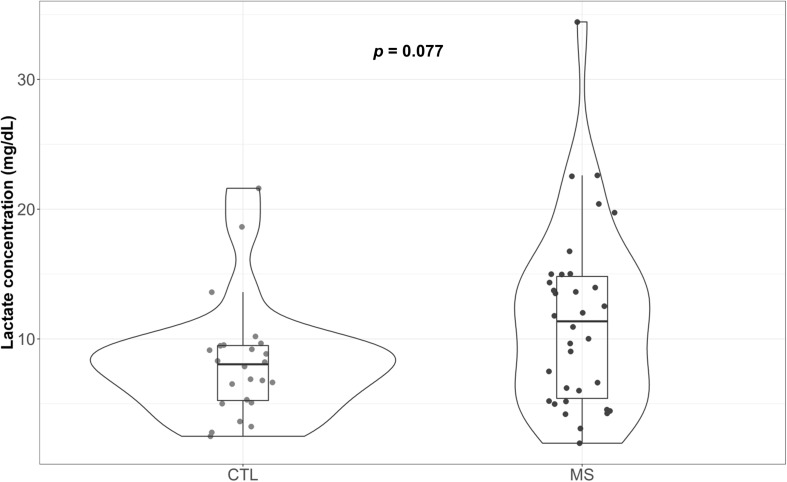
Violin plot and boxplot of plasma lactate concentration attributable to each group. Concentration of plasma lactate determined in blood samples.

Figure 3 illustrate the circulating plasma lactate detected in MS patients and controls. Median lactate concentration was 8.04 mg/dL (5.25, 9.49) for the control group. Although not significant, a 41% increase in this value was detected for MS patients [11.36 mg/dL (5.41, 14.81)]. While the means of both groups were within the range of standard concentrations established for the healthy population (between 4.5 and 19.8 mg/dL plasma), note that four (11.8%) of the patients included in the MS group exceeded the maximum levels, reaching values of 20.40, 22.53, 22.61, and 34.43 mg/dL. In the control group, however, only one patient exceeded that limit (4.2%). When results were adjusted for sex and age, the reported increase was statistically significant (*p* = 0.013) (Supplementary Table S1).

### MS Patients Exhibit Reduced Plasma Antioxidant Capacity

**FIGURE 4:**
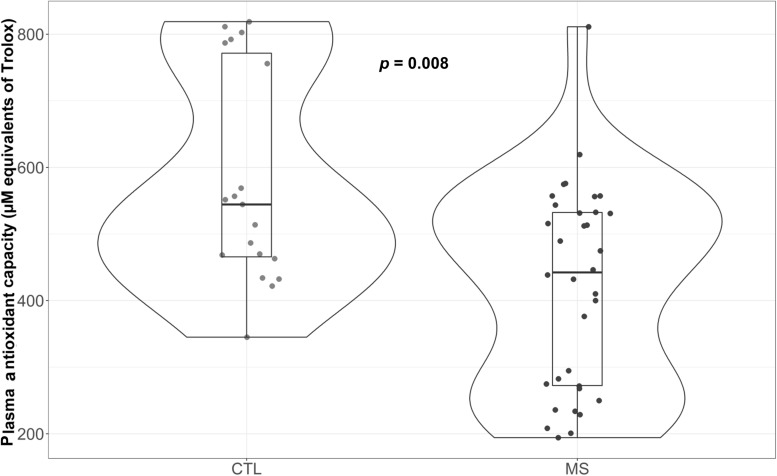
Violin plot and boxplot of antioxidant capacity measured as μM equivalents of Trolox. MS patients show lower plasma antioxidant capacity.

As can be observed in Figure 4, MS patients exhibited reduced antioxidant capacity (*p* = 0.008) compared to controls. Moreover, this difference was maintained when results were adjusted for sex, age, and uric acid (*p* = 0.006) (Supplementary Table S1). Since variations in several metabolites could explain these changes (Re et al., 1999), we evaluated the major component of antioxidant capacity in plasma and uric acid. Results revealed no significant differences in this parameter between control group and plasma samples from MS patients (data not shown).

## Discussion

To our knowledge, this is the first functional study to explore the redox status of peripheral lymphocytes from MS patients. We found signs of OS in these cells together with an impaired antioxidant capacity that could be contributing to this phenomenon.

Thus, our results demonstrate, for the first time, that the peripheral lymphocytes of MS patients produce higher concentrations of superoxide compared to healthy subjects. This supports the mitochondrial involvement in MS pathogenesis previously reported by other studies. As examples, a proper axonal mitochondrial content has been shown to be directly related to the remyelination process (Zambonin et al., 2011) and mitochondrial dysfunction in CNS cells has been linked to axonal degeneration (Dutta et al., 2006; Su et al., 2009; Campbell et al., 2014). However, mitochondrial studies in peripheral lymphocyte populations have been very limited to date. Our data demonstrate that peripheral T lymphocytes present higher levels of the superoxide anion, pointing to involvement of these cells in the pathophysiology of the disease. Although further research is required, these cells might represent a potential therapeutic target for the early stages of MS. Notably, results are consistent when they are adjusted for cell count, sex and age. This is important since these variables may influence the antioxidant capacity and consequently modify the results, which is not the case.

The decreased levels of mitochondrial respiratory complex proteins reported in our study support the abovementioned hypothesis, suggesting mitochondrial involvement in MS ethiopathology. More importantly, these results may be indicative of possible impaired function of mitochondrial complexes, which could explain the abnormal superoxide production reported here, especially taking into account that both complexes I and III are major intracellular sources of this anion (Boveris and Chance, 1973; Forman and Boveris, 1982) (the complexes whose differences are also maintained after the adjustment). Besides, genetic studies have demonstrated an imbalance in the expression of mitochondrial complex encoding genes in postmortem brain samples of MS patients (Pandit et al., 2009). Specifically, the decreased expression of those gens in the CNS cells of MS models is reported (Dutta et al., 2006; Pandit et al., 2009), suggesting that certain variations in the content of these complexes might be caused by the disease. Our data may also support these results. The decreased levels of complexes I and III in peripheral blood lymphocytes of MS patients might also be an indicator of the subsequent events occurring in the CNS, since when activated, these cells have the ability to cross the BBB (Abbas et al., 2012), and might transfer their mitochondrial mismatch to the parenchyma, increasing ROS production. Considering that T lymphocytes are in part responsible for the inflammatory process typical of MS, treatments aimed at limiting the infiltration of these cells could reduce not only the inflammation, but also the concomitant CNS OS, one of the potential causes of neurodegeneration (Lee et al., 2014). Further studies are required to explore the use of this parameter as a biomarker of disease progression, with possible clinical benefit even for patients who experience clinical or radiologic isolated syndrome. For these patients, this marker might be used as a risk indicator for MS development, representing a benefit for early treatment.

Mitochondrial complexes work together but, as in all enzyme systems, do not work continuously at full capacity. Consequently, it is tempting to hypothesize that, since lymphocytes have a lower content of complex I and III, these complexes should operate at a higher capacity. This would lead to lower efficiency and higher electron leakage, which might explain the increased superoxide production. Our results would support the notion of this increased superoxide production in part due to a mismatch in mitochondrial complexes I and III. Additionally, this would lead to an increase in total ROS levels which, in turn, could result in early depletion of the antioxidant defenses in the plasma and, consequently, to a diminished antioxidant capacity as reported here. On the other hand, the existence of a higher order of mitochondrial structures: supercomplexes (Lenaz et al., 2016), would add a layer of complexity to the relationship between superoxide production and mitochondrial changes. This would relate decreased levels of complexes I and III to decreased ATP synthesis (Lenaz et al., 2016) and, as a consequence, ATP synthase (complex V) expression would be reduced, as we have described here. These results could suggest a new pathological mechanism for this heterogeneous disease, which might help to explain the lack of therapeutic response experienced by some patients (patients with suboptimal response to treatment). Furthermore, our results may help to identify potential therapeutic targets, as well as biomarkers of clinical activity that could be useful as diagnostic and prognostic tools for targeted and specific treatments.

Analysis of the lactate concentration, a potential proxy for mitochondrial respiration loss leading to anaerobic glycolysis, revealed significantly increased levels in the plasma of MS patients when we adjust results for potential confounders such as age and sex. Our results contrast with those described by Mähler et al. (2012) in which no significant differences were observed in the plasma lactate concentration between healthy individuals and MS patients. However, another study conducted in a much larger cohort (*n* = 1238) showed that serum lactate concentration could be a powerful biomarker in MS (Amorini et al., 2014). A metabolic switch at systemic level could explain our results, but the exact causes are still unknown. A possible explanation is that part of this increase could be due to mitochondrial changes in the amounts of complex I and III, in line with the finding that specific lymphocytes have the ability to activate the fermentation of pyruvate to lactate (Jones and Thompson, 2007). However, it is worth noting that there is a substantial contribution of skeletal muscle to circulating lactate, which may explain why MS patients with an increased fatigue burden due to lack of mobility exhibit high plasma lactate levels. In contrast, a significant correlation between lactate production and EDSS was not found (data not shown), although other authors have reported this finding in plasma (Amorini et al., 2014) [but not in CSF (Regenold et al., 2008)]. In any case, we cannot rule out that part of the increase in plasma lactate may be due to the limitation of mitochondrial respiration.

Increased superoxide production could be directly related to a buildup of cellular oxidative damage (Cadenas, 1989), potentially in line with increased plasma oxidative modifications, as suggested by an increase in markers of oxidative damage both in plasma (unpublished data) and CSF (Gonzalo et al., 2012). Although the increase in plasma OS markers in MS is well documented (Koch et al., 2007; Karlík et al., 2015; Pasquali et al., 2015), the origin of this damage remains unclear. It has been suggested that these phenomena could originate in the CNS and, since in MS the BBB is compromised, these could spread systemically.

The changes in the redox status of peripheral lymphocytes from MS patients described here might partially explain the OS phenomenon in the CNS reported in this disease. According to our hypothesis, T cells would act as a Trojan horse, transporting peripherally originated OS to the CNS.

However, this study has some limitations. First, the cross-sectional design of the study is a major limitation, since it does not allow causal relationships to be established. Secondly, we would like to point out that the sample size of the present study might be low to perform multivariable analysis. Nonetheless, we have used multivariable linear regression to estimate the effect associated with MS while controlling potential biases due to confounding factors.

Despite these limitations, we believe that this study provides unique and interesting fresh data that shed new light on the role of OS and mitochondrial (dys) function in MS. Of note, one of the first antibodies used in MS was Natalizumab, binding to lymphocytes very late antigen 4 and preventing lymphocytes from crossing the blood-brain barrier, thereby improving clinical course (Polman et al., 2006). The same basic mechanism, i.e., inhibiting the entry of T cells in CNS, is shared by Fingolimod and ozanimod acting on sphingosine-1-phosphate cellular responses (Cohen et al., 2010, 2016). Besides avoiding the above-referred trojan-horse effect of high-superoxide producing T cells infiltrating CNS, it is known that α4 integrin, target of natalizumab, mediates free radical-derived damage peripherally (Poon et al., 2001). Though this is speculative, perhaps the effects of natalizumab on T cells also involve diminished free radical production. Supporting this, it is known that natalizumab diminishes OS biomarkers in MS patients (Tasset et al., 2013). In line with this, recent data show that polymorphisms in genes encoding oxidative detoxification components, such as NQO1 and GSTP1, are relevant in the therapeutic response to this drug (Alexoudi et al., 2016). Recent data also show that sphingosine-1-phosphate inhibition prevents oxidative-damage in glial cells (O’Sullivan et al., 2017). Noteworthy, one of major targets of Sphingosine-1-phosphate is mitochondrial function of naive T cells (Mendoza et al., 2017). Our finding of deregulated mitochondrial function in T cells could be related to these novel effects of sphingosine-1-phosphate in T cells. Last, but not least, dimethyl fumarate treatments are clearly targeted to several factors evaluated here, such as mitochondrial function and oxidative damage, among others (Kees, 2013; Peruzzotti-Jametti and Pluchino, 2018). In this sense, recent data show that dimethyl fumarate, acting in neurodegenerative models is able to modulate NRF2 signaling (Ranea-Robles et al., 2018). Of note, NRF2 is a key factor modulating responses to OS, even involving changes in mitochondrial function and cellular metabolism (Holmström et al., 2013). Therefore, dimethyl fumarate clinical effects might involve modulatory effects on mitochondrial dysfunction hereby described in MS. To sum up, we believe that the association between MS treatment and T-cell superoxide overproduction and mitochondrial (dys) function are worth exploring in future studies. Further, our data support the potential use of mitochondrial characteristics of circulating T cells for development of biomarkers of disease activity, and they may contribute to heterogeneity to treatment response and MS prognosis.

## Conclusion

Our findings demonstrate that PBMCs of MS patients show impaired mitochondrial redox status and deficient antioxidant capacity. These results allow us to speculate that peripheral immune cells might partially contribute to the OS reported in the CNS of MS patients.

## Data Availability

All datasets generated for this study are included in the manuscript and/or the Supplementary Files.

## Ethics Statement

Human Subject Research: The studies involving human participants were reviewed and approved by the Arnau de Vilanova University Hospital (Lleida, Spain). The patients/participants provided their written informed consent to participate in this study.

## Author Contributions

LN, HG, AG-S, and JH performed all the experimental measurements. MM-G, SP, and MS were responsible for sample collection and pre-processing. CG-M and LB conducted the neurological examinations of MS patients with explaining the project and collection of informed consent. MC and MP-O were responsible for performing the statistical analyses. RP and JS assisted with the data interpretation and article design. LN and HG contributed equally in planning the experimental work, preparing figures, performing general analyses, and writing the manuscript. All the team has been working under the coordination, supervision, and direction of LB.

## Conflict of Interest Statement

The authors declare that the research was conducted in the absence of any commercial or financial relationships that could be construed as a potential conflict of interest.

## References

[B1] Abbas A. K. Andrew H. L. Pillai S. (2012). *Inmunología Celular y Molecular.* Madrid: W.B. Saunders.

[B2] Alexoudi A. Zachaki S. Stavropoulou C. Gavrili S. Spiliopoulou C. Papadodima S. (2016). Possible implication of GSTP1 and NQO1 polymorphisms on natalizumab response in multiple sclerosis. *Ann. Clin. Lab. Sci.* 46 586–591. 27993870

[B3] Amorini A. M. Nociti V. Petzold A. Gasperini C. Quartuccio E. Lazzarino G. (2014). Serum lactate as a novel potential biomarker in multiple sclerosis. *Biochim. Biophys. Acta* 1842 1137–1143. 10.1016/j.bbadis.2014.04.005 24726946

[B4] Ascherio A. Munger K. L. (2010). Epstein-barr virus infection and multiple sclerosis: a review. *J. Neuroimmune Pharmacol.* 5 271–277. 10.1007/s11481-010-9201-3 20369303

[B5] Back S. A. Han B. H. Luo N. L. Chricton C. A. Xanthoudakis S. Tam J. (2002). Selective vulnerability of late oligodendrocyte progenitors to hypoxia-ischemia. *J. Neurosci.* 22 455–463. 10.1523/JNEUROSCI.22-02-00455.2002 11784790 PMC6758669

[B6] Boveris A. Chance B. (1973). The mitochondrial generation of hydrogen peroxide. General properties and effect of hyperbaric oxygen. *Biochem. J.* 134 707–716. 10.1042/bj1340707 4749271 PMC1177867

[B7] Cadenas E. (1989). Biochemistry of oxygen toxicity. *Annu. Rev. Biochem.* 58 79–110. 10.1146/annurev.biochem.58.1.79 2673022

[B8] Cai Z. Xiao M. (2016). Oligodendrocutes and Alzheimer’s disease. *Int. J. Neurosci.* 126 97–104. 26000818 10.3109/00207454.2015.1025778

[B9] Campbell G. R. Worrall J. T. Mahad D. J. (2014). The central role of mitochondria in axonal degeneration in multiple sclerosis. *Mult. Scler.* 20 1806–1813. 10.1177/1352458514544537 25122475

[B10] Chastain E. M. Miller S. D. (2012). Molecular mimicry as an inducing trigger for CNS autoimmune demyelinating disease. *Immunol. Rev.* 245 227–238. 10.1111/j.1600-065X.2011.01076.x 22168423 PMC3586283

[B11] Cohen J. A. Arnold D. L. Comi G. Bar-Or A. Gujrathi S. Hartung J. P. (2016). Safety and efficacy of the selective sphingosine 1-phosphate receptor modulator ozanimod in relapsing multiple sclerosis (RADIANCE): a randomised, placebo-controlled, phase 2 trial. *Lancet Neurol.* 15 373–381. 10.1016/S1474-4422(16)00018-1 26879276

[B12] Cohen J. A. Barkhof F. Comi G. Hartung H. P. Khatri B. O. Montalban X. (2010). Oral fingolimod or intramuscular interferon for relapsing multiple sclerosis. *N. Engl. J. Med.* 362 402–415. 10.1056/NEJMoa0907839 20089954

[B13] Corthay A. (2006). A three-cell model for activation of naïve T helper cells. *Scand. J. Immunol.* 64 93–96. 10.1111/j.1365-3083.2006.01782.x 16867153

[B14] Cunniffe N. Coles A. (2019). Promoting remyelination in multiple sclerosis. *J. Neurol.* 10.1007/s00415-019-09421-x [Epub ahead of print]. 31190170 PMC7815564

[B15] Dutta R. McDonough J. Yin X. Peterson J. Chang A. Torres T. (2006). Mitochondrial dysfunction as a cause of axonal degeneration in multiple sclerosis patients. *Ann. Neurol.* 59 478–489. 10.1002/ana.20736 16392116

[B16] Dyment D. A. Ebers G. C. Dessa Sadovnick A. (2004). Genetics of multiple sclerosis. *Lancet Neurol.* 3 104–110. 14747002 10.1016/s1474-4422(03)00663-x

[B17] Fiorini A. Koudriavtseva T. Bucaj E. Coccia R. Foppoli C. Giorgi A. (2013). Involvement of oxidative stress in occurrence of relapses in multiple sclerosis: the spectrum of oxidatively modified serum proteins detected by proteomics and redox proteomics analysis. *PLoS One* 8:e65184. 10.1371/journal.pone.0065184 23762311 PMC3676399

[B18] Forman H. J. Boveris A. (1982). “Superoxide radical and hydrogen peroxide in mitochondria,” in *Free Radicals in Biology*, ed. Pryor W. (New York, NY: Academic Press).

[B19] French H. M. Reid M. Mamontov P. Simmons R. A. Grinspan J. B. (2009). Oxidative stress disrupts oligodendrocyte maturation. *J. Neurosci. Res.* 87 3076–3087. 10.1002/jnr.22139 19479983 PMC3138415

[B20] Fünfschilling U. Supplie L. M. Mahad D. Boretius S. Saab A. S. Edgar J. (2012). Glycolytic oligodendrocytes maintain myelin and long-term axonal integrity. *Nature* 485 517–521. 10.1038/nature11007 22622581 PMC3613737

[B21] Gilgun-Sherki Y. Melamed E. Offen D. (2004). The role of oxidative stress in the pathogenesis of multiple sclerosis: the need for effective antioxidant therapy. *J. Neurol.* 251 261–268. 10.1007/s00415-004-0348-9 15015004

[B22] Gonzalo H. Brieva L. Tatzber F. Jove M. Cacabelos D. Cassanye A. (2012). Lipidome analysis in multiple sclerosis reveals protein lipoxidative damage as a potential pathogenic mechanism. *J. Neurochem.* 123 622–634. 10.1111/j.1471-4159.2012.07934.x 22924648

[B23] Haider L. (2015). Inflammation, iron, energy failure, and oxidative stress in the pathogenesis of multiple sclerosis. *Oxid. Med. Cell Longev.* 2015:725370. 10.1155/2015/725370 26106458 PMC4461760

[B24] Halliwell B. Gutteridge J. M. C. (2007). *Free Radicals in Biology and Medicine*, 4th Edn New York, NY: Oxford University Press.

[B25] Hewer S. Lucas R. van der Mei I. Taylor B. V. (2013). Vitamin D and multiple sclerosis. *J. Clin. Neurosci.* 20 634–641. 10.1016/j.jocn.2012.10.005 23540892

[B26] Holmström K. M. Baird L. Zhang Y. Hargreaves I. Chalasani A. Land J. M. (2013). Nrf2 impacts cellular bioenergetics by controlling substrate availability for mitochondrial respiration. *Biol. Open* 2 761–770. 10.1242/bio.20134853 23951401 PMC3744067

[B27] Jäger A. Dardalhon V. Sobel R. A. Bettelli E. Kuchroo V. K. (2009). Th1, Th17, and Th9 effector cells induce experimental autoimmune encephalomyelitis with different pathological phenotypes. *J. Immunol.* 183 7169–7177. 10.4049/jimmunol.0901906 19890056 PMC2921715

[B28] Jones R. G. Thompson C. B. (2007). Revving the engine: signal transduction fuels T cell activation. *Immunity* 27 173–178. 10.1016/j.immuni.2007.07.008 17723208

[B29] Karlík M. Valkovič P. Hančinová V. Krížová L. Tóthová L′. Celec P. (2015). Markers of oxidative stress in plasma and saliva in patients with multiple sclerosis. *Clin. Biochem.* 48 24–28. 10.1016/j.clinbiochem.2014.09.023 25304914

[B30] Kees F. (2013). Dimethyl fumarate: a Janus-faced substance? *Expert Opin. Pharmacother.* 14 1559–1567. 10.1517/14656566.2013.804912 23697607

[B31] Koch M. Mostert J. Arutjunyan A. V. Stepanov M. Teelken A. Heersema D. (2007). Plasma lipid peroxidation and progression of disability in multiple sclerosis. *Eur. J. Neurol.* 14 529–533. 10.1111/j.1468-1331.2007.01739.x 17437612

[B32] Kroenke M. A. Carlson T. J. Andjelkovic A. V. Segal B. M. (2008). IL-12- and IL-23-modulated T cells induce distinct types of EAE based on histology, CNS chemokine profile, and response to cytokine inhibition. *J. Exp. Med.* 205 1535–1541. 10.1084/jem.20080159 18573909 PMC2442630

[B33] Larsson N.-G. (2010). Somatic mitochondrial DNA mutations in mammalian aging. *Annu. Rev. Biochem.* 79 683–706. 10.1146/annurev-biochem-060408-093701 20350166

[B34] Lee J. Y. Taghian K. Petratos S. (2014). Axonal degeneration in multiple sclerosis: can we predict and prevent permanent disability? *Acta Neuropathol. Commun.* 2:97. 10.1186/s40478-014-0097-7 25159125 PMC4243718

[B35] Lenaz G. Tioli G. Falasca A. I. Genova M. L. (2016). Complex I function in mitochondrial supercomplexes. *Biochim. Biophys. Acta* 1857 991–1000. 10.1016/j.bbabio.2016.01.013 26820434

[B36] Mahad D. Ziabreva I. Lassmann H. Turnbull D. (2008). Mitochondrial defects in acute multiple sclerosis lesions. *Brain* 131 (Pt 7) 1722–1735. 10.1093/brain/awn105 18515320 PMC2442422

[B37] Mahad D. J. Ziabreva I. Campbell G. Lax N. White K. Hanson P. S. (2009). Mitochondrial changes within axons in multiple sclerosis. *Brain* 132 (Pt 5) 1161–1174. 10.1093/brain/awp046 19293237 PMC3605917

[B38] Mähler A. Steiniger J. Bock M. Brandt A. U. Haas V. Boschmann M. (2012). Is metabolic flexibility altered in multiple sclerosis patients? *PLoS One* 7:e43675. 10.1371/journal.pone.0043675 22952735 PMC3429505

[B39] Marui N. Offermann M. K. Swerlick R. Kunsch C. Rosen C. A. Ahmad M. (1993). Vascular cell adhesion molecule-1 (VCAM-1) gene transcription and expression are regulated through an antioxidant-sensitive mechanism in human vascular endothelial cells. *J. Clin. Invest.* 92 1866–1874. 10.1172/jci116778 7691889 PMC288351

[B40] Mendoza A. Fang V. Chen C. Serasinghe M. Verma A. Muller J. (2017). Lymphatic endothelial S1P promotes mitochondrial function and survival in naive T cells. *Nature* 546 158–161. 10.1038/nature22352 28538737 PMC5683179

[B41] Miljković D. Spasojević I. (2013). Multiple sclerosis: molecular mechanisms and therapeutic opportunities. *Antioxid. Redox Signal.* 19 2286–2334. 10.1089/ars.2012.5068 23473637 PMC3869544

[B42] Murray J. R. Rajeevan M. S. (2013). Evaluation of DNA extraction from granulocytes discarded in the separation medium after isolation of peripheral blood mononuclear cells and plasma from whole blood. *BMC Res. Notes* 6:440. 10.1186/1756-0500-6-440 24176175 PMC3818442

[B43] Nogueras L. Gonzalo H. Jové M. Sol J. Gil-Sanchez A. Hervás J. V. (2019). Lipid profile of cerebrospinal fliuid in multiple sclerosis patients: a potential tool for diagnosis. *Sci. Rep.* 9:11313. 10.1038/s41598-019-47906-x 31383928 PMC6683197

[B44] Nylander A. Hafler D. A. (2012). Multiple sclerosis. *J. Clin. Invest.* 122 1180–1188. 10.1172/JCI58649 22466660 PMC3314452

[B45] Ohl K. Tenbrock K. Kipp M. (2016). Oxidative stress in multiple sclerosis: central and peripheral mode of action. *Exp. Neurol.* 277 58–67. 10.1016/j.expneurol.2015.11.010 26626971 PMC7094520

[B46] Oliveira S. R. Kallaur A. P. Simão A. N. Morimoto H. K. Lopes J. Panis C. (2012). Oxidative stress in multiple sclerosis patients in clinical remission: association with the expanded disability status scale. *J. Neurol. Sci.* 321 49–53. 10.1016/j.jns.2012.07.045 22883481

[B47] Olsen N. K. Hansen A. W. Norby S. Edal A. L. Jorgensen J. R. Rosenberg T. (1995). Leber’s hereditary optic neuropathy associated with a disorder indistinguishable from multiple sclerosis in a male harbouring the mitochondrial DNA 11778 mutation. *Acta Neurol. Scand.* 91 326–329. 10.1111/j.1600-0404.1995.tb07016.x 7639060

[B48] O’Sullivan S. A. Velasco-Estevez M. Dev K. K. (2017). Demyelination induced by oxidative stress is regulated by sphingosine 1-phosphate receptors. *Glia* 65 1119–1136. 10.1002/glia.23148 28375547

[B49] Pandit A. Vadnal J. Houston S. Freeman E. McDonough J. (2009). Impaired regulation of electron transport chain subunit genes by nuclear respiratory factor 2 in multiple sclerosis. *J. Neurol. Sci.* 279 14–20. 10.1016/j.jns.2009.01.009 19187944

[B50] Pasquali L. Pecori C. Lucchesi C. LoGerfo A. Iudice A. Siciliano G. (2015). Plasmatic oxidative stress biomarkers in multiple sclerosis: relation with clinical and demographic characteristics. *Clin. Biochem.* 48 19–23. 10.1016/j.clinbiochem.2014.09.024 25300461

[B51] Pender M. P. Burrows S. R. (2014). Epstein-Barr virus and multiple sclerosis: potential opportunities for immunotherapy. *Clin. Transl. Immunol.* 3:e27. 10.1038/cti.2014.25 25505955 PMC4237030

[B52] Peruzzotti-Jametti L. Pluchino S. (2018). Targeting mitochondrial metabolism in neuroinflammation: towards a therapy for progressive multiple sclerosis. *Trends Mol. Med.* 24 838–855. 10.1016/j.molmed.2018.07.007 30100517

[B53] Polman C. H. O’Connor P. W. Havrdova E. Hutchinson M. Kappos L. Miller D. H. (2006). A randomized, placebo-controlled trial of natalizumab for relapsing multiple sclerosis. *N. Engl. J. Med.* 354 899–910. 16510744 10.1056/NEJMoa044397

[B54] Polman C. H. Reingold S. C. Banwell B. Clanet M. Cohen J. A. Filippi M. (2011). Diagnostic criteria for multiple sclerosis: 2010 revisions to the McDonald criteria. *Ann. Neurol.* 69 292–302. 10.1002/ana.22366 21387374 PMC3084507

[B55] Poon B. Y. Ward C. A. Cooper C. B. Giles W. R. Burns A. R. Kubes P. J. (2001). alpha(4)-integrin mediates neutrophil-induced free radical injury to cardiac myocytes. *Cell Biol.* 152 857–866. 10.1083/jcb.152.5.857 11238444 PMC2198813

[B56] Ranea-Robles P. Launay N. Ruiz M. Calingasan N. Y. Dumont M. Naudí A. (2018). Aberrant regulation of the GSK-3β/NRF2 axis unveils a novel therapy for adrenoleukodystrophy. *EMBO Mol. Med.* 10:e8604. 10.15252/emmm.201708604 29997171 PMC6079538

[B57] Re R. Pellegrini N. Proteggente A. Pannala A. Yang M. Rice-Evans C. (1999). Antioxidant activity applying an improved ABTS radical cation decolorization assay. *Free Radic. Biol. Med.* 26 1231–1237. 10.1016/s0891-5849(98)00315-3 10381194

[B58] Regenold W. T. Phatak P. Makley M. J. Stone R. D. Kling M. A. (2008). Cerebrospinal fluid evidence of increased extra-mitochondrial glucose metabolism implicates mitochondrial dysfunction in multiple sclerosis disease progression. *J. Neurol. Sci.* 275 106–112. 10.1016/j.jns.2008.07.032 18783801 PMC2584157

[B59] Reich D. S. Lucchinetti C. F. Calabresi P. A. (2018). Multiple Sclerosis. *N. Engl. J. Med.* 378 169–180. 29320652 10.1056/NEJMra1401483 PMC6942519

[B60] Ridet J. L. Malhotra S. K. Privat A. Gage F. H. (1997). Reactive astrocytes: cellular and molecular cues to biological function. *Trends Neurosci.* 20 570–577. 10.1016/s0166-2236(97)01139-9 9416670

[B61] Robinson K. M. Janes M. S. Pehar M. Monette J. S. Ross M. F. Hagen T. M. (2006). Selective fluorescent imaging of superoxide in vivo using ethidium-based probes. *Proc. Natl. Acad. Sci. U.S.A.* 103 15038–15043. 10.1073/pnas.0601945103 17015830 PMC1586181

[B62] Ruiz-Argüelles A. Pérez-Romano B. (2001). Immunophenotypic analysis of peripheral blood lymphocytes. *Curr. Protoc. Cytom.* Chapter 6:Unit 6.5. 10.1002/0471142956.cy0605s11 18770716

[B63] Sadovnick A. D. Armstrong H. Rice G. P. Bulman D. Hashimoto L. Paty D. W. (1993). A population-based study of multiple sclerosis in twins: update. *Ann. Neurol.* 33 281–285. 8498811 10.1002/ana.410330309

[B64] Salim S. (2017). Oxidative stress and the central nervous system. *J. Pharmacol. Exp. Ther.* 360 201–205. 27754930 10.1124/jpet.116.237503 PMC5193071

[B65] Sospedra M. Martin R. (2005). Immunology of multiple sclerosis. *Annu. Rev. Immunol.* 23 683–747. 15771584 10.1146/annurev.immunol.23.021704.115707

[B66] Su K. G. Banker G. Bourdette D. Forte M. (2009). Axonal degeneration in multiple sclerosis: the mitochondrial hypothesis. *Curr. Neurol. Neurosci. Rep.* 9 411–417. 10.1007/s11910-009-0060-3 19664372 PMC2839873

[B67] Tasset I. Bahamonde C. Agüera E. Conde C. Cruz A. H. Pérez-Herrera A. (2013). Effect of natalizumab on oxidative damage biomarkers in relapsing-remitting multiple sclerosis. *Pharmacol. Rep.* 65 624–631. 10.1016/s1734-1140(13)71039-9 23950585

[B68] Toshniwal P. K. Zarling E. J. (1992). Evidence for increased lipid peroxidation in multiple sclerosis. *Neurochem. Res.* 17 205–207. 10.1007/bf00966801 1538834

[B69] Turrens J. F. (2003). Mitochondrial formation of reactive oxygen species. *J. Physiol.* 552 (Pt 2) 335–344. 10.1113/jphysiol.2003.049478 14561818 PMC2343396

[B70] Wallace D. C. Singh G. Lott M. T. Hodge J. A. Schurr T. G. Lezza A. M. (1988). Mitochondrial DNA mutation associated with Leber’s hereditary optic neuropathy. *Science* 242 1427–1430. 3201231 10.1126/science.3201231

[B71] Wu M. Tsirka S. E. (2009). Endothelial NOS-deficient mice reveal dual roles for nitric oxide during experimental autoimmune encephalomyelitis. *Glia* 57 1204–1215. 10.1002/glia.20842 19170181 PMC2706940

[B72] Yakes F. M. Van Houten B. (1997). Mitochondrial DNA damage is more extensive and persists longer than nuclear DNA damage in human cells following oxidative stress. *Proc. Natl. Acad. Sci. U.S.A* 94 514–519. 10.1073/pnas.94.2.514 9012815 PMC19544

[B73] Zambonin J. L. Zhao C. Ohno N. Campbell G. R. Engeham S. Ziabreva I. (2011). Increased mitochondrial content in remyelinated axons: implications for multiple sclerosis. *Brain* 134 (Pt 7) 1901–1913. 10.1093/brain/awr110 21705418 PMC3122369

