# Supplementary material for: Impairment of Mitochondrial Redox Status in Peripheral Lymphocytes of Multiple Sclerosis Patients
Source: Front Neurosci. 2019 Sep 4;13:938. doi: 10.3389/fnins.2019.00938 (PMC6738270; doi:10.3389/fnins.2019.00938)
Supplement: Supplementary file 1 [file Table_1.DOCX]

**Supplementary table**

**Table S1**. Adjusted differences between MS patients and controls by means of multivariate linear regression.

| Study outcome | Adjusted mean difference^a^, MS vs CTL (95% CI) | *p*-value^b^ |
| --- | --- | --- |
| Total superoxide, MFI | 330.9 (17.4, 644.4) | 0.044 |
| T-cell superoxide, MFI | 148.7 (-21.6, 318.9) | 0.094 |
| B-cell superoxide, MFI | 133.4 (-23.4, 290.2) | 0.102 |
| Monocyte superoxide, MFI | 29.8 (-3.3, 63) | 0.084 |
| MC-I, AU | -1.48 (-2.72, -0.25) | 0.026 |
| MC-II, AU | -1.13 (-2.99, 0.72) | 0.240 |
| MC-III, AU | -1.99 (-3.73, -0.24) | 0.033 |
| MC-IV, AU | -0.80 (-2.48, 0.87) | 0.356 |
| MC-V, AU | -1.94 (-3.65, -0.24) | 0.033 |
| Lactate concentration, mg/dL | 4.07 (0.98, 7.16) | 0.013 |
| Antioxidant plasma capacity, μM Trolox equivalents | -128.8 (-216.6, -41) | 0.006 |
| a. All mean differences were adjusted for sex and age. Superoxide mean differences were additionally adjusted for cell count. Antioxidant plasma capacity mean difference was additionally adjusted for uric acid concentration.  b. P-values correspond to the t test for significance testing of multivariate linear model parameters.  **MS** = multiple sclerosis group, **CTL** = control group, **CI** = confidence interval, **MFI** = mean fluorescence intensity, **MC** = mitochondrial complex, **AU** = arbitrary units. | | |
